# A Case of Anomalous Hysteria

**Published:** 1843-11-11

**Authors:** Robert H. Cummins

**Affiliations:** Wheeling, Va.


### A CASE OF ANOMALOUS HYSTERIA.

BY ROBERT H. CUMMINS, M. D., OF WHEELING, VA.

Miss ——, aged twenty-three years, was attacked about twenty months since, with pains of a neuralgic character, extending over the sides and breast, and down the arms. They were preceded by a few days sickness, which, so far as I could learn, consisted in derangement of the stomach and bowels. A dose of calomel had been administered, and this produced salivation. The pains were so severe as to occasion loud screams, which could be heard in the neighboring houses, and they were constant in the day time, but intermitted during the night. About the same period every evening they would suddenly cease, and from a condition of the greatest distress, the patient would fall into a pleasant sleep. In the morning, generally a short time after daybreak, she would be roused by a return of the pain, and a shriek usually announced its accession to her friends. She remained in this condition for probably two months, tortured by pain during the day, but enjoying a respite in the night.

A new symptom then made its appearance. During the day-time the patient began to have intermissions of pain. This of itself would rather have been hailed as a favorable symptom; but when we discovered that the intermissions were attended with complete insensibility, it excited our alarm. She would suddenly cease to moan, and her countenance, from an expression of the greatest agony, would change to one of perfect quietude. Her friends would familiarly say she had a *sick spell.* These intervals of ease would last from five minutes to half an hour. Their termination would be announced by a groan, and at the same instant the pain would return. They would occur sometimes three or four times in a day, and sometimes as often as twenty, and I observed that in proportion to their frequency, was the severity of the pain. When in this state she was insensible to all external impressions. Her eyes would be closed, and the lids would sometimes tremble: at other times they would give only an occasional twitch. Upon opening them the eye-ball had a normal appearance —the pupil was natural in size, and the color looked healthy. If you spoke to her she either did not hear, or she was unable to make any answer, for no reply ever escaped her. You might pinch her or pull her, still she gave no sign of sensibility. Whilst in this condition all the functions of animal life were suspended, and those of organic life were more or less impeded. The pulse would frequently become weak and fluttering, and the respiration, though generally quiet and easy, would sometimes be hurried or interrupted by sighs.

During the whole of our patient's sickness, she had no appetite. In a day she would often not eat four ounces of food, and this she would force upon herself rather as a means of sustenace, than because she had any relish for it.

Her bowels, by the aid of occasional doses of laxative medicine, were kept regular, and her stools had generally a healthy appearance. Her tongue would often become coated, and the coating was usually of a yellow color. The condition of her tongue, stomach, and bowels seemed to have no influence over the pain. Their derangement rather seemed consequential to it.

The catamenial function was performed during the whole disease regularly, and differed in no respect, so far as was observable, from its condition in health.

The urinary and perspiratory discharges were carried on with due regularity. That from the skin occasionally emitted an unpleasant smell, and the odour was peculiar, for I never smelt any thing either before or since, like it. It somewhat resembled that which arises from a dead body.

At times she had an irresistible desire to laugh, and generally this was succeeded by crying, and increased severity of pain. On several occasions she begged us to hold her hands, for she said if she got an opportunity, she would certainly bite her fingers off. This was a mere fancy, however, for though she often succeeded in getting them into her mouth, she never had the courage to bring the blood.

A slight review of the symptoms easily determined the character of the pain, and our remedial agents were, of course, directed to the nervous symptoms.

The treatment was commenced by the use of anodynes and anti-spasmodics. The whole range of these divisions of the materia medica was gone over, without any permanent benefit resulting. Opiates had the effect of lulling the pain for a time, but as soon as their stupefying influence wore off, it would again return, and oftentimes with augmented violence. After taking any preparation of opium (and every variety was tried), she always complained of uneasy sensations in the head, and these were so unpleasant that she herself preferred not using it. Valerian, castor, musk, aud assafœtida were tried in all their various forms of preparation, without any benefit.

Tonics were employed, but even those from which we were led to expect most advantage, proved unavailing. Iron, combined with anti-spasmodics, in form of Vallet's proto-carbonate, and the common precipitated carbonate: quinine, and the other vegetable bitters were each tried, but all with the same unfavorable result. From the analogy that subsisted between the pain and that of tic douloureux, I was induced to try the power of Fowler's solution of arsenite of potash, but this likewise failed to exercise any influence over the disease.

Our medication, heretofore, had all been based upon the supposition that the disease was merely a functional derangement of the nervous system. And indeed all the symptoms of the case seemed to indicate this, for upon the closest examination no tenderness could be discovered along the spine, and our patient was at all times free from fever. It was determined, notwithstanding, to try the effects of counter irritation, and the back was accordingly rubbed with tartar emetic ointment, from the nape of the neck to the sacrum. This was kept discharging for two months without producing the least effect, in the relief of pain. It did not even mitigate it. In connection an application of veratria, in form of solution, was made to the sides, breast and arms; and this, notwithstanding its reputed efficacy in allaying neuralgic pain, done no good.

The case had now run on for about four months, and there was not the least abatement in the symptoms. The patient was suffering extreme agony, and the friends began justly to entertain fears that she could not survive. In this dilemma I felt like abandoning the case, for I could think of no other means that afforded a reasonable prospect of relief.

It may be stated that the advice of other physicians was obtained: and, whilst all concurred in the propriety of what had been done, none recommended other means that were likely to do good.

I had suggested the use of hydrocyanic acid to other physicians who had seen the case, but it never received the sanction of any one. Its administration was thought to be attended with danger without affording any prospect of benefit. I was, nevertheless, from a knowledge of its powerful influence over the nervous system, induced to try it. I accordingly gave Scheele's preparation in the dose of one-twelfth of a drop. This made a powerful and unexpected impression on her system. It threw her into a condition precisely similar to the state we designated a *sick spell*, with the exception, only, that all the symptoms were aggravated. She became nearly pulseless, sighed frequently, and was insensible. I tried in vain to arouse her. This spell was protracted so long as to render it alarming. It continued during the whole night and until the next evening, embracing a period of about twenty-four hours. From the commencement of this attack I found it necessary to stimulate her, and wine was administered pretty freely. I likewise had sinapisms applied to her feet and arms, and I kept cold applications on her head. She was at length roused, and from that moment she began to mend. She was troubled no more with pain: after a few days her appetite returned, and she gained strength rapidly.

The prussic acid in this case relieved the pain, but why it operated with so much violence I am unable to say. Probably the diseased condition of her nervous system was of such a nature as to render her peculiarly susceptible to its action.

During the space of about six months our patient enjoyed uninterrupted good health. She had improved in health and appearance, and felt at no time any return of pain.

At the expiration of this period, however, the pain returned in great violence, and with the suddenness of thought. She was pursuing some one of her ordinary occupations, and had to drop it instantly and scream. I was called to her, and though I felt confident prussic acid would relieve her, I was deterred from again administering it. I feared its effects. For two months she suffered extreme pain, alternated every day with *sick spells*, as they were familiarly called, and during this time she again went the rounds of the anodynes and anti-spasmodics. They afforded no relief.

The friends applied to a quack doctor in the neighborhood, and he gave her a great variety of *nerve drops*, as he termed them, to take inwardly, and *embrocations* to rub externally, but all to no purpose.

In deliberating further on this case, it was suggested to my mind to give tartar emetic in nauseating doses, and I was induced to try it in the expectation that it would have a favorable revulsive effect, or that a change would be made in the disease, through the intervention of the nausea produced. I accordingly gave it, and had the satisfaction to find that it effected all I desired. As soon as nausea was produced the pain abated, and under its continuance it vanished entirely. It is now seven months since, and our patient has had no return, but she has been improving ever since. If the pain should return, I feel assured that the tartar emetic will control it at any time, and perhaps any other nauseating medicine would have the same effect.

After the pain subsided it was many days before Miss —— recovered her appetite. Her stomach seemed obstinately deranged. It, however, got better at length, and her health now seems in every respect as good as ever it was.

